# Selective Detection of Active Extracellular Granzyme A by Using a Novel Fluorescent Immunoprobe with Application to Inflammatory Diseases

**DOI:** 10.1021/acsptsci.4c00065

**Published:** 2024-04-22

**Authors:** Ana Senan-Salinas, Laura Comas, Patricia Esteban, Marcela Garzón-Tituaña, Zhiming Cheng, Llipsy Santiago, Maria Pilar Domingo, Ariel Ramírez-Labrada, José Ramón Paño-Pardo, Marc Vendrell, Julián Pardo, Maykel A. Arias, Eva M. Galvez

**Affiliations:** † Instituto de Carboquímica ICB-CSIC, 50018 Zaragoza, Spain; ‡ Fundación Instituto de Investigación Sanitaria Aragón (IIS Aragón), Biomedical Research Centre of Aragón (CIBA), 50009 Zaragoza, Spain; § Dept. Microbiology, Preventive Medicine and Public Health, University of Zaragoza, 50009 Zaragoza, Spain; ∥ CIBERINFEC, ISCIII—CIBER de Enfermedades Infecciosas, Instituto de Salud Carlos III, 28029Madrid, Spain; ⊥ Servicio de Enfermedades Infecciosas, Hospital Clinico Universitario Lozano Blesa, 50009 Zaragoza, Spain; # Centre for Inflammation Research, The University of Edinburgh, EH164UU Edinburgh, U.K.; ∇ Unidad de Nanotoxicología e Inmunotoxicología (UNATI), Centro de Investigación Biomédica de Aragón (CIBA), Aragón Health Research Institute (IIS Aragón), 50009Zaragoza, Spain; ○ IRR Chemistry Hub, Institute for Regeneration and Repair, The University of Edinburgh, EH16 4UU Edinburgh, U.K.

**Keywords:** GzmA activity, probe, fluorescence, inflammatory disease

## Abstract

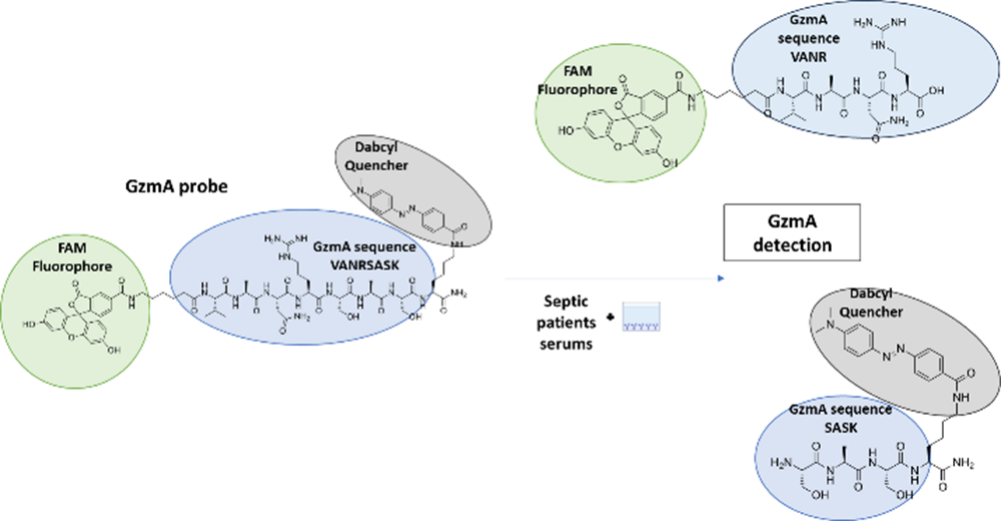

Granzymes (Gzms), a family of serine proteases, expressed by immune and nonimmune cells, present perforin-dependent and independent intracellular and extracellular functions. When released in the extracellular space, GzmA, with trypsin-like activity, is involved in the pathophysiology of different inflammatory diseases. However, there are no validated specific systems to detect active forms of extracellular GzmA, making it difficult to assess its biological relevance and potential use as a biomarker. Here, we have developed fluorescence-energy resonance-transfer (FRET)-based peptide probes (FAM-peptide-DABCYL) to specifically detect GzmA activity in tissue samples and biological fluids in both mouse and human samples during inflammatory diseases. An initial probe was developed and incubated with GzmA and different proteases like GzmB and others with similar cleavage specificity as GzmA like GzmK, thrombin, trypsin, kallikrein, or plasmin. After measuring fluorescence, the probe showed very good specificity and sensitivity for human and mouse GzmA when compared to GzmB, its closest homologue GzmK, and with thrombin. The specificity of this probe was further refined by incubating the samples in a coated plate with a GzmA-specific antibody before adding the probe. The results show a high specific detection of soluble GzmA even when compared with other soluble proteases with very similar cleavage specificity like thrombin, GzmK, trypsin, kallikrein, or plasmin, which shows nearly no fluorescence signal. The high specific detection of GzmA was validated, showing that using pure proteins and serum and tissue samples from GzmA-deficient mice presented a significant reduction in the signal compared with WT mice. The utility of this system in humans was confirmed, showing that GzmA activity was significantly higher in serum samples from septic patients in comparison with healthy donors. Our results present a new immunoprobe with utility to detect extracellular GzmA activity in different biological fluids, confirming the presence of active forms of the soluble protease in vivo during inflammatory and infectious diseases.

Granzymes (Gzms) are a family of serine proteases expressed in immune and nonimmune cells that are classified according to their cleavage specificity. In humans, five Gzms have been described (A, B, H, K, and M), while 10 have been described in mice (A, B, C, D, E, F, G, K, M, and N). However, GzmA and GzmB are the most abundant and still best characterized.^1^ Together with perforin, Gzms constitute the main component of the granule exocytosis pathway.^2,3^ This is a specialized form of intracellular protein delivery, by which cytotoxic T (Tc) and natural killer (NK) cells release Gzms into the cytosol of target cells through perforin-mediated membrane pores. Once in the cytosol Gzms will induce different biological functions including cell death and pathogen inactivation.^3−5^ On the other hand, Gzms can also be released into the extracellular milieu where they will exert different perforin-independent extracellular functions^4^ mainly related to extracellular matrix degradation and regulation of inflammation.^4^ Extracellular GzmB has been involved in wound healing, skin aging, coagulation, and endothelial cell barrier integrity.^6,7^ On the other hand, extracellular GzmA has been shown to activate inflammation in different cell types^8−12^ and has been involved in several inflammatory and autoimmune diseases including endotoxicosis, sepsis, arthritis, colitis, and inflammatory colorectal cancer.^9,12−15^ Although increased levels of soluble GzmA have been detected in biological fluids of human inflammatory or infectious diseases,^4,16,17^ it is not clear yet whether the active forms of soluble GzmA are released during these pathologies. This is challenging since, based on its cleavage specificity, GzmA belongs to the family of trypsin proteases that includes the closest homologues of GzmA, GzmK, thrombin, trypsin, plasmin, or kallikrein, all of which are proteases that, similar to GzmA, can be present in extracellular fluids and cleave similar peptide sequences as GzmA. Albeit extracellular protease activity is tightly regulated by the presence of extracellular inhibitors to avoid tissue damage, it is well-known that either an increase in the protease levels or a decrease of the inhibitor levels occurs physiologically to promote different functions like coagulation, cell migration, or tissue repair.^18,19^ However, dysregulation of this balance will lead to the pathological function of these proteases in different diseases. Thus, the detection of the active forms of these proteases like GzmA would not only confirm its role in inflammatory diseases but, in addition, allow the use of it as a biomarker for disease monitoring.

Several sensors have been developed to detect protease activity in biological fluids based on FRET assays that present a high sensitivity.^20^ These sensors often consist of a short peptide linked to a donor and an acceptor in close proximity; thus, the emission of the fluorescence donor is quenched by the acceptor. When the protease cleaves the peptide substrate, it will cause the FRET pair to separate and the fluorescence signal to increase.^21,22^ However, specific probes or biosensors capable of discriminating GzmA from its closest homologues of the trypsin family have not been previously validated, and thus, at present it is not possible to reliably detect soluble GzmA activity in inflammatory and autoimmune diseases with high morbidity and mortality.

In this work, we have developed a fluorescent peptide-based probe that allows selective detection of GzmA activity in comparison with GzmB, GzmK, and thrombin and further using it to develop an immunoprobe based on the peptide and a GzmA-specific antibody for highly specific detection of GzmA, differentiating it from its closest homologues trypsin, plasmin, and kallikrein. These probes have been validated in vitro and in vivo using different samples including cell/tissue lysates and fluids from WT and GzmA^KO^ animals and human samples from healthy donors and septic patients, supporting its application to detect active forms of GzmA in both human and mouse systems.

## Materials and Methods

### Reagents, Proteins, and ELISA

The DNA sequences encoding amino acid residues of the mouse and human GzmA were synthesized by GenScript (USA) for expression in *Pichia pastoris* using the protein expression vector pPICZαA, called pPICZαA-m/hGzmA. Both plasmids contain at the 5′ end a two-residue amino acid sequence encoding for the GzmA inactive zymogen. pPICZαA-GzmA were linearized by SacI-HF overnight at 37 °C and transformed by electroporation into X-33 yeast strain, clones were selected on YPD plates containing 200 μg/mL of zeocin (InvivoGen, USA) and colonies were grown in BMGY medium overnight at 30 °C in a shaking incubator (250 rpm). Batch yeast cells were harvested by centrifugation and resuspended in BMMY medium to induce expression at room temperature for 72–96 h with shaking at 250 rpm. Methanol was added every 24 h to a final concentration of 1% v/v. The supernatants containing soluble secreted zymogens (pro-Gzms) of GzmA were filtered using 0.45 and 0.22 μm filters and concentrated to 20–40 mL using a PelliconTM XL Biomax 10 kDa (Merck-Millipore, Germany). Subsequently, GzmA was dialyzed against buffer A (25 mM MES pH6) and loaded into a 1 × 5 mL His-Trap Column (GE Healthcare, USA) previously equilibrated on an AKTA purifier system. Following loading, the column was washed with buffer B (25 mM MES pH 6, 150 mM NaCl) and proteins were eluted using a NaCl gradient (0 to 1M) in buffer A. Fractions containing GzmA were pooled, and buffer exchanged into buffer C (150 mM NaCl, 4 mM NaH_2_PO_4_, 16 mM Na_2_HPO_4_; pH7.5) using a PD-10 desalting column (GE Healthcare, USA). The eluted fractions were concentrated and stored at −80 °C until used for the experiments. Pro-GzmA was activated by incubation with Cathepsin C (Sigma, USA) in buffer C supplemented with 0.2 M sodium acetate, pH 5.5. GzmA activity of every stock was assessed by hydrolysis of the commercial substrates Bz-Pro-Phe-Arg-pNA-HCl (Bz-PFR-pNA; BACHEM, Switzerland) for mGzmA and Z-Gly-Pro-Arg-pNA acetate salt (Z-GPR-pNA; BACHEM, Switzerland) for hGzmA in enzyme-specific buffer (100 mM TRIS pH8.5) at 37 °C as previously described.^7^

Human GzmK and GzmB were purchased from Enzo Life Sciences (USA). Thrombin, plasmin, trypsin, and kallikrein were purchased from Merck Millipore/Sigma-Aldrich (Germany).

A mouse GzmA homemade ELISA has been previously described.^14^

### Mouse Strains

Inbred C57Bl/6JRccHsd (B6) and mouse strains deficient for GzmA (GzmA^–/–^) and GzmK (GzmK^–/–^), bred on B6 background, were maintained at the Centre for Biomedical Research of Aragon (CIBA), and analyzed for their genotypes as described.^23^ Mice from both sexes and 8–10 weeks of age were used in all experiments that were performed in accordance with FELASA guidelines under the supervision and approval of the Ethics Committee for Animal Experimentation from the University of Zaragoza (number: PI64/17).

### Mouse Infection with LCMV and *Escherichia coli*

Mice were infected with 10^5^ pfu of **LCMV** strain WE intraperitoneally (i.p.) according to the established protocols.^24^ On day 8 postinfection, the time at which the peak of cellular immune response is reached, blood samples were collected from mice by the technique of cardiac puncture, and plasma was recovered by centrifugation at 3700 × *g*. Tissues (spleen and liver) were isolated and homogenized in RIPA buffer according to the manufacturer’s instructions (Sigma, USA). CD8^+^ cells were positively selected from spleens using α-CD8-MicroBeads (MiltenyiBiotec, Germany) with MACS (MiltenyiBiotec, Germany) and resuspended in RIPA lysis buffer. The purity of selected CD8^+^ cells was assessed by FACS staining and was found to be between 95 and 98% in all cases. All samples were stored at −80 °C before using in cleavage assays.

An *E. coli* strain previously isolated from the blood of mice suffering from abdominal sepsis^25^ was used. Inoculum for sepsis induction was prepared by culturing *E. coli* stock in LB medium at 37 °C to the exponential growth phase and washed twice with cold phosphate-buffered saline (PBS). The absorbance at 600 nm was measured to estimate the number of bacteria in the culture. Bacterial density was adjusted to 1 × 10^9^ bacteria/ml and sepsis was induced in mice by i.p. injection of 2 × 10^8^ bacteria in PBS.^25^ After 24 h, plasma samples were obtained and stored as described above.

### Patients

A total of 19 healthy donors and 28 patients with a diagnosis of abdominal sepsis were prospectively recruited for hospital admission at University Hospital Lozano Blesa in Zaragoza, Spain. Blood samples (26) and peritoneal lavage samples (2) were collected. Blood samples were spun down at 2000 × *g* for 10 min and serum was stored at −80 °C. The procedure was previously approved by the Clinical Ethics Committee of Aragon (CEICA) under number PI18/023

### HPLC-MS Analysis

The enzymatic cleavage was monitored by HPLC-MS using an HPLC Agilent Technologies 1200 with a Kinetex C18 50 × 4.6 mm column and a diode array detector. Eluents: H_2_O (0.1% HCOOH) and ACN (0.1% HCOOH). Flow: 1.0 mL min^–1^. The MS detector was configured with an electrospray ionization source, and nitrogen was used as the nebulizer gas.

### FRET Assays

Two potential GzmA protease substrates based either on short peptide screening, VANRSAS^26^ (probe-1) or in a natural substrate cleavage sequence, EDMAKSDKAR^27^ (probe 2) were synthesized with a fluorescent donor at the amino (N-) terminal, carboxyfluorescein (FAM), and a fluorescent quencher at the carboxyl (C−) terminal, 4-(4-dimethylaminophenylazo)benzoic acid (DABCYL), by Pepscan (Switzerland). The probe is 96.1% pure according to its supplier. The rest of the compounds are commercial; therefore, their purity is guaranteed by their providers.

Measurement of quenching efficiency was determined using a 1:1 donor/acceptor ratio at the optimal excitation and emission wavelengths (Ex_max_ = 415 nm, Em_max_ = 517 nm) and calculated by dividing fluorescence intensity in the presence and absence of 1 μM quencher according to the following equation:
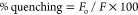
*F*_o_: fluorescence in the quenched state. *F*: fluorescence in the activated state.

The fluorescence was measured in a FluoroMax-P spectrofluorometer (Horiba), and data were analyzed using the software DataMax.

Analysis of proteolytic activity with FRET probes was performed using different concentrations of pure proteins from the same batch at 37 °C in a thermostatic microplate shaker. Assays were performed using 1 μM substrate in a final volume of 100 μL in enzyme-specific buffer (100 mM TRIS-HCl pH 8.5) in 96-well plates. FAM dequenching was monitored at 475–517 nm for the indicated times. A negative control reaction was also performed using the same reaction with an inactive precursor (pro-GzmA) or without enzymes. A trypsin 0.05% solution (Gibco, Thermo Fisher Scientific, USA) was used as a positive control. Experiments were repeated at least three times.

In order to normalize the activity of the proteases used in the in vitro FRE*T* assays with probe-1, the specific proteolytic activity of every protease was quantified with the general trypsin-like protease substrate Nα-CBZ-l-Lysine thiobenzyl ester hydrochloride. The experiment was performed as indicated above for the specific GzmA substrates, but in this case, the substrate was previously activated with 5,5′-dithio-bis(2-nitrobenzoic acid). In the case of GzmB presenting aspartase activity, the specific substrate Ac-Ile-Glu-Pro-Asp-pNA (Ac-IEPD-pNA) was used.

Activity assays were carried out to analyze the specificity of every protease (hGzmA, mGzmA, hGzmK, hGzmB, thrombin, plasmin, kallikrein, and trypsin) for probe-1 and to calculate the kinetic constants of the proteases that cleaved probe-1.

The kinetics were analyzed by incubating the proteases with probe-1 at 37 °C for 24 h in a final volume of 200 ul in the enzyme-specific buffer. The concentrations of proteases were 66.7 nM hGzmA, 4 nM mGzmA, 3.7 nM kallikrein, 4.3 nM plasmin, 0.4 nM trypsin, 16.8 nM thrombin, 2.4 nM hGzmK, and 0.2 U/μL hGzmB corresponding to the same enzyme activities calculated as indicated above. The peptide concentration was 1 μM. Fluorescence was measured every 5 min during the first hour, every 15 min during the second hour, every 30 min the following two h, and finally, every hour up to 24 h as indicated above. The specific increases at 4 and 24 h were calculated by subtracting the values at time 0 from the 4 and 24 h values, respectively.

Those proteases that cleaved probe-1 were used to calculate the kinetic constants. They were incubated with different probe-1 concentrations (from 20 to 0.16 μM) using the same enzyme units except hGzmA which was diluted to 16.68 nM. The total volume of the reaction was 100 μL in enzyme-specific buffer, and fluorescence was measured in a 96-well plate reader (BioTek Synergy HT) as indicated above. To get an accurate result on kinetic constants, the measurement was made every 5 min during 1.5 h at 37 °C. Fluorescence units were transformed into activity values by extrapolating on a FAM calibration curve. These activity values were plotted against time, resulting in different curves for each substrate. The slope of these curves in their initial linear part was the reaction velocity (*V*_o_). Vo values were plotted against substrate concentrations and the *K*_M_ and *V*_max_ parameters were calculated using Michaelis–Menten nonlinear regression in GraphPad.

### Protease Activity in Biological Samples

After the selection of optimum substrate and incubation time, 10 μL of different tissue lysates and serum samples were mixed with 1 μM probe-1 at 37 °C in 96-well plates (100 μL/well) and incubated for 1 h using an enzyme-specific buffer. Fluorescence was monitored at 475 and 520 nm. Experiments were repeated at least twice.

### Analysis of Protease Activity Using a Combination of Anti-GzmA Antibodies and Probe-1

For selective detection of hGzmA, a combination of a specific hGzmA mAb with FRET probe-1 was used. Commercial plates with precoated hGzmA mAb were used (Thermo Fisher, USA). Different concentrations of pure proteases and samples (sera and peritoneal lavages) from septic patients were incubated for 2 h at RT. After washing the plate with buffer (1X PBS, 0.05% Tween 20) the FRET probe was added at 1 μM and incubated at 37 °C. Fluorescence was measured after 1, 2, 4, 20, and 24 h of incubation as indicated above. In the case of mGzmA, the protocol was similar, but in this case, we used a rabbit antimouse GzmA to coat plates at 2.5 μg/mL in coating buffer (1X PBS) for 24 h at 4 °C.

### Statistical Analysis

Statistical analyses were performed using GraphPad software, version 7. The difference between means of unpaired samples was performed using a two-way analysis of variance with Dunnet’s post-test or using unpaired *t*-test as indicated in the legend of each figure. The difference between the inhibition percentages was performed by a two-way ANOVA test. In all cases, a statistically significant difference was determined as * *p* < 0,05; ** *p* < 0,01; *** *p* < 0,001; ****p < 0,0001.

## Results

### Peptide Substrate Design

**Figure 1:**
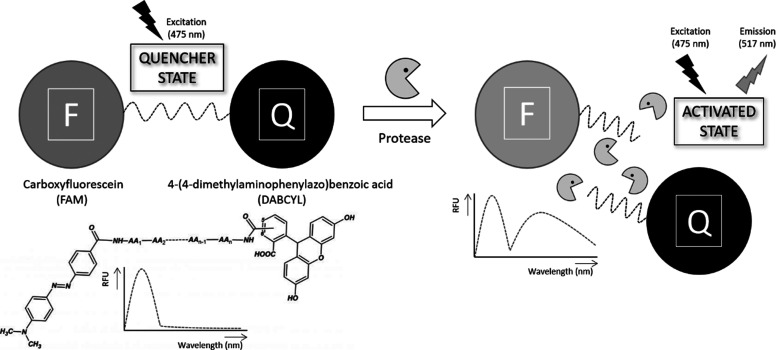
Possible states of peptide depending on the donor–acceptor distance. When the quencher DABCYL and the fluorophore FAM are close in the same peptide, the fluorescence emitted by FAM is quenched by DABCYL. However, if a protease cuts the specific peptide sequence, the distance of quencher and fluorophore increases, so the quenching effect disappears and FAM fluorescence emission is detected. Hence, we can find two possible states: “quenched” and “active”.

We selected 2 peptides based on the potential sequences present in short peptides and natural substrates that most likely could be cleaved by human and mouse GzmA,^26−28^ and designed two quenching fluorescence resonance energy transfer (FRET)-based probes using FAM as the donor and the quencher DABCYL as the acceptor (Figure 1).

### Efficiency, Sensitivity, and Specificity of Probes

**Figure 2:**
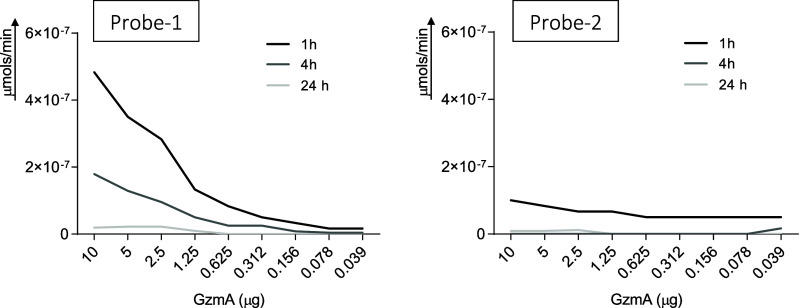
Sensitivity of probe-1 and probe-2 against mGzmA. Different concentrations (from 10 to 0.039 ng) of recombinant mGzmA were incubated with probe-1 and probe-2 both at 1 μM. After 1, 4, and 24 h of incubation, the fluorescence intensity was measured (λ_exc_ = 475 nm, λ_em_ = 520 nm). At each of the values obtained, the signal of peptide alone was subtracted. Enzyme activity values were calculated by extrapolating fluorescence units on a FAM calibration curve. Data are presented as mean of three independent experiments.

First of all, we analyzed the ability of mouse GzmA to cleave probe-1 and probe 2. As shown in Figure 2, probe-1 was able to detect enzymatic activity until 78 ng of mGzmA after 1 h of incubation. After 4 and 24 h incubation, the sensitivity decreased, detecting 0.625 and 1.250 μg of mGzmA, respectively. In contrast, probe-2 detected similar enzymatic activity for different concentrations of mGzmA after 1 h of incubation, and after 4 h and after 24 h of incubation probe-2 was not capable of detecting mGzmA enzymatic activity at any concentration.

**Figure 3:**
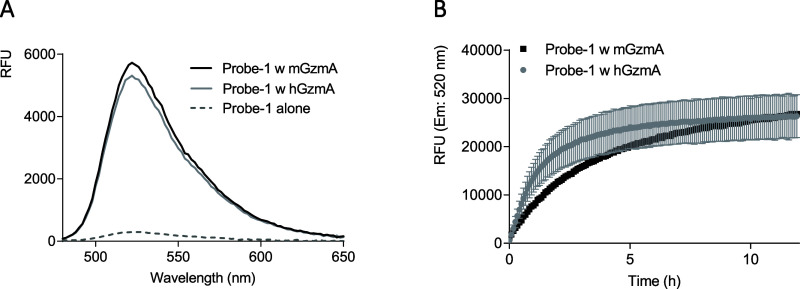
Probe-1 detects both mouse and human GzmA activity. (A) Emission spectra of the probe-1 alone (20 μM) or after incubation with hGzmA or mGzmA (20 nM) at 37 °C for 16 h. (B) Kinetics of the fluorescence intensity of probe-1 (20 μM) after incubation with hGzmA or mGzmA (20 nM).

Based on these results, we selected probe-1 to further analyze its potential as a GzmA-specific detection system and analyzed the different parameters to evaluate the performance of such a probe using both human and mouse GzmA. The sensitivity of probe-1 for hGzmA and mGzmA was analyzed. First, we analyzed the kinetics of probe-1 cleavage by mGzmA or hGzmA. The emission of probe-1 was analyzed in the absence or presence of GzmA. As expected, the peptide alone emits almost no signal at 520 nm, but the peptide after incubation with GzmA emits a great fluorescence signal (Figure 3A), being slighter higher when using mGzmA in comparison with hGzmA. Confirming this result, detailed kinetics analyses during 12 h showed that probe-1 was more efficiently cleaved by mGzmA than hGzmA (Figure 3B), albeit in both cases a remarkable fluorescence signal was detected, indicating that probe-1 is useful for detecting GzmA activity in both mice and humans

**Figure 4:**
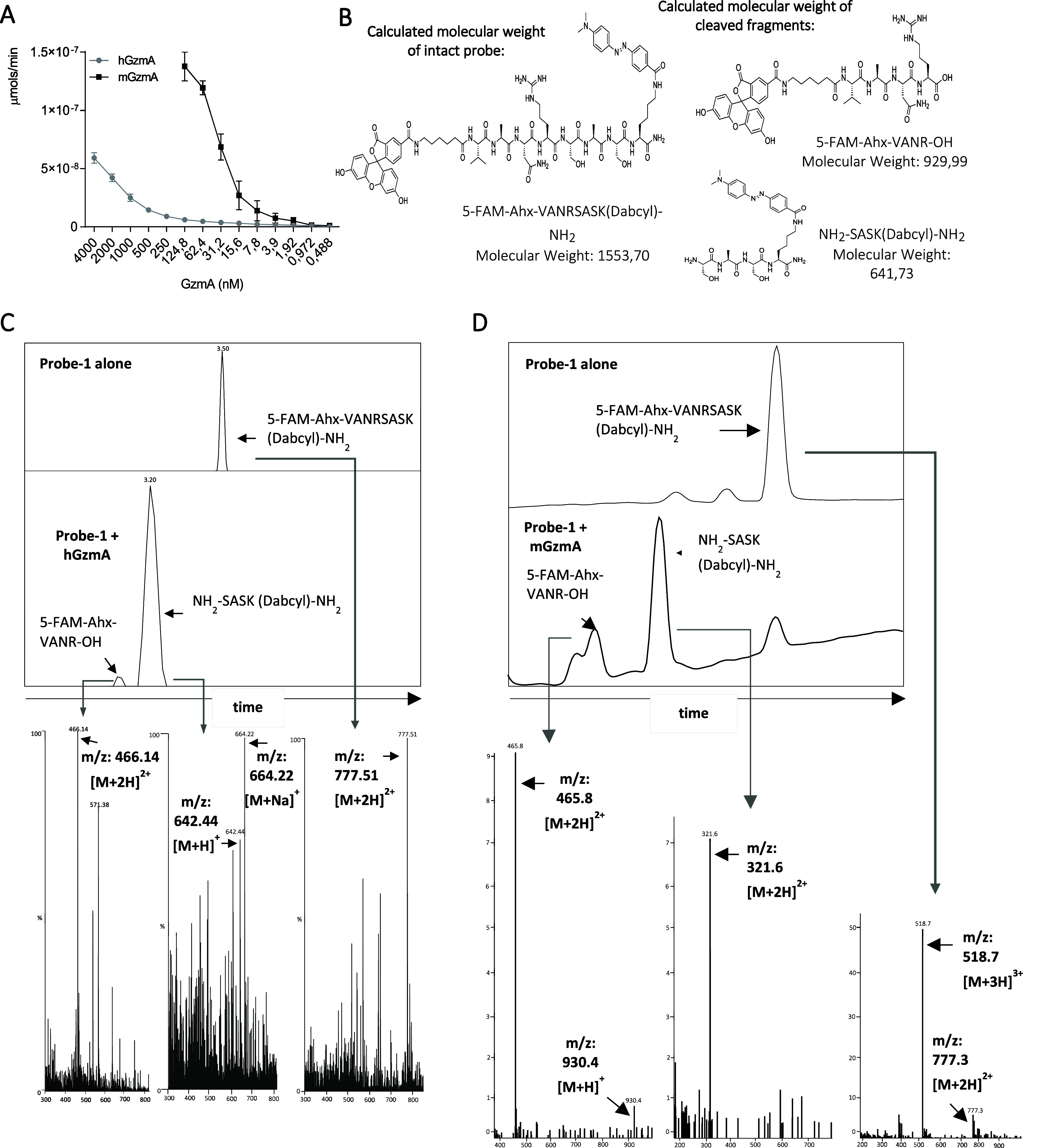
Sensitivity of probe-1 for human and mouse GzmA. (A) Different concentrations (from 4000 nM to 0,488 nM) of recombinant mGzmA and hGzmA were incubated with probe-1. After 1 h, the fluorescence intensity was measured (λ_exc_ = 475 nm, λ_em_ = 520 nm) and transformed into enzyme activity value calculated by extrapolating fluorescence units on a FAM calibration curve. Data are presented as the mean ± SEM of three independent experiments. (B) Theoretical molecular weight of the intact probe-1 and of the cleaved peptide fragments. (C) Probe-1 alone (20 μM) and incubated with hGzmA (20 nM) at 37 °C for 16 h was analyzed by HPLC-MS. (D) Same experiment as that in B was performed but using mGzmA (20 nM).

Next, the sensitivity was calculated by incubating different concentrations of GzmA with probe-1 for 1 h. As shown in Figure 4A, the sensitivity for mGzmA was higher than that for hGzmA independently of the concentration used. Detection limits were 0.972 and 3.9 nM for mGzmA and hGzmA, respectively.

To confirm that probe-1 was specific toward GzmA and cleavage occurred at the expected site (VANR↓S), the enzymatic reaction was monitored using HPLC-MS. As shown in Figure 4B–D, probe-1 alone resulted in a single peak, whereas after incubation with either human (Figure 4C) or mouse (Figure 4D) GzmA, two clear peaks were detected, whose respective masses corresponded with the expected cleaved fragments.

### Specificity of Probe-1 for GzmA and Other Proteases

A few previous studies have developed fluorescent probes to detect intracellular GzmA activity, mainly in human cells.^29−31^ However, the specificity of these probes was mainly assessed against proteases that were not closely related to GzmA in terms of cleavage specificity. Thus, we decided to determine whether probe-1 was able to selectively detect GzmA in comparison with the closest homologues to GzmA regarding cleavage specificity (trypsin-like activity), which could be expressed in cells, serum, or other biological fluids. The serine proteases selected were hGzmA, hGzmK mGzmA, plasmin, thrombin, kallikrein, and trypsin. In addition, we included GzmB as a negative control as one of the main members of the Gzm family. In order to perform a meaningful comparison, we needed to use the same amount of enzymatic units, thus, the activity of the different trypsin-like proteases was equated using a common substrate, Nα-CBZ-l-lysine thiobenzyl ester hydrochloride, as indicated in methods (Supp. Figure 1).

**Figure 5:**
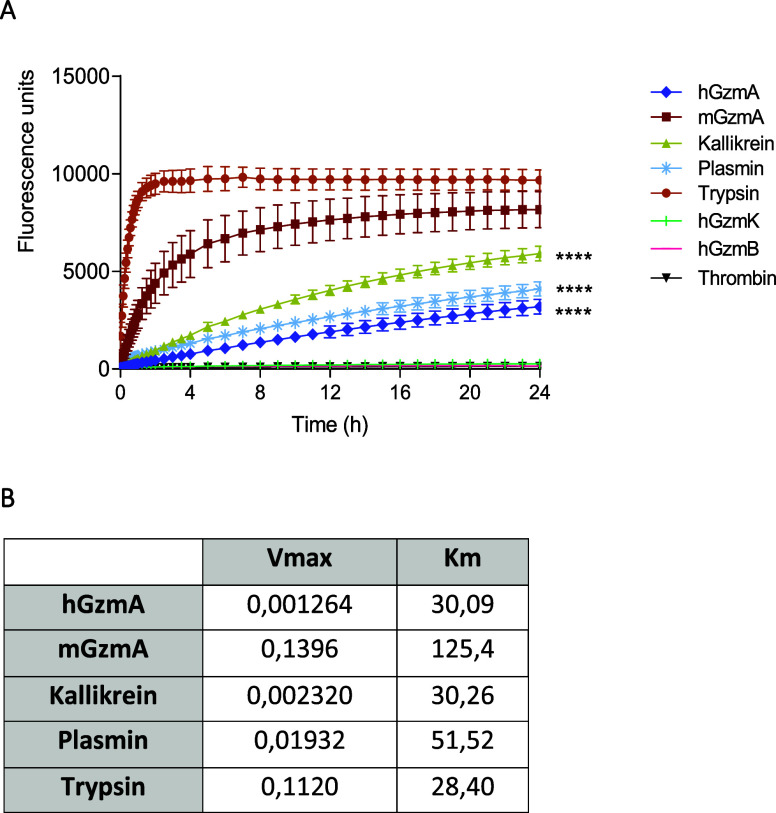
Kinetics of different proteases with probe-1. (A) Probe-1 (1 μM) was incubated with the same EU of each protease (calculated as indicated in methods) in 100 mM TRIS-HCl buffer pH 8.5. The fluorescence intensity (λ_exc_ = 475 nm, λ_em_ = 520 nm) was measured at 37 °C for 24 h. The values obtained at each time were subtracted from the signal of the peptide in the absence of enzymes. The curves represent the mean ± SEM of 3 independent experiments. Statistical analyses were performed by the two-way ANOVA test with Bonferroni’s post-test. ****p < 0.0001. (B) Different concentrations of probe-1 (from 20 to 0.16 μM) were incubated with the different proteases: hGzmA (16.68 nM), mGzmA (4 nM), kallikrein (3.7 nM), plasmin (4.3 nM), and trypsin (0.4 nM). The fluorescence intensity (λ_exc_ = 475 nm, λ_em_ = 520 nm) was measured every 5 min for 90 min. At each of the values obtained, the peptide signal in the absence of enzymes was subtracted. Km and Vmax parameters were calculated in GraphPad Prism by using Michaelis–Menten nonlinear regression.

As shown in Figure 5A, trypsin and mGzmA efficiently cleaved probe-1 reaching the highest fluorescent after approximately 2 and 12 h, respectively. Probe-1 was cleaved more slowly and similarly by kallikrein, plasmin, and hGzmA, and the highest fluorescent intensity was not reached even after 24 h. Probe-1 was not cleaved by hGzmK and thrombin or GzmB. Significant differences were found when comparing trypsin with the other proteases, which cleave probe-1 except for mGzmA.

Next, we calculated the kinetic constants of every protease using the same EU and different concentrations of probe-1 using Michaelis–Menten nonlinear regression. As shown in Figure 5B, hGzmA has the lowest Vmax and a very low Km, indicating a high affinity for the peptide. However, mGzmA is the protease with higher values of *K*_m_ and *V*_max_, so it is the enzyme with less affinity for substrate. Other proteases such as kallikrein, plasmin, and trypsin have similar kinetic constants, so their affinity for probe-1 is comparable.

### Analysis of Probe Specificity Using Samples from WT and KO Mice

**Figure 6:**
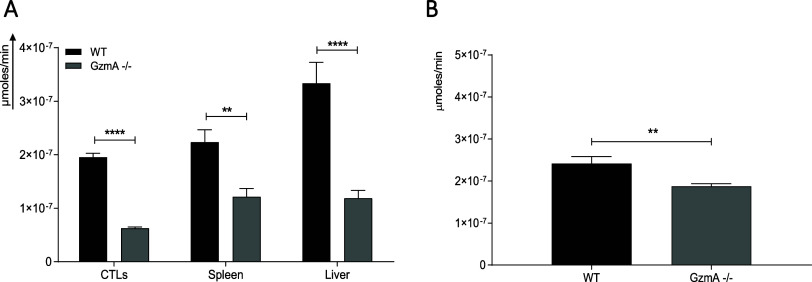
GzmA activity in tissue/cell lysates and serum samples of WT and GzmA^KO^mice infected with LCMV or *E. coli*. C57Bl/6 (B6) WT or GzmA^KO^ mice were inoculated with 10^5^ pfu of the LCMV WE strain or with 2 × 10^8^ CFU of *E. coli* intraperitoneally. (A) After 8 days, mice were sacrificed and spleens and livers were collected and frozen. In parallel, CD8^+^T cells were enriched from splenocytes as indicated in the Materials and Methods. Lysate supernatants were prepared as indicated in Materials and Methods, and GzmA activity was monitored after 1 h of incubation with probe-1. (B) After 24 h of sepsis induction, mice were sacrificed, and serum samples were collected. The fluorescence intensity (λ_exc_ = 475 nm, λ_em_ = 520 nm) was measured after 1 h. At each of the values obtained, the signal of samples without probe-1 and probe-1 without samples were subtracted. Data are represented as the mean values ± SEM of at least three different mice from two independent experiments. Statistical analysis was performed using unpaired *t* test. ** *p* < 0.01; ****p < 0.0001; ns, not significant.

The selectivity of probe-1 was also analyzed using lysate supernatants from different tissues and cells from WT and GzmA^KO^ mice infected with LCMV that increased GzmA expression. As shown in Figure 6A, the activity of GzmA was significantly higher in WT than in GzmA^KO^ mice in all samples assayed, confirming a good selectivity of probe-1 to detect GzmA in ex vivo-derived biological samples.

The probe-1 was further validated by analyzing serum of septic WT and GzmA^KO^ mice infected with *E. coli*.

As shown in Figure 6B, there was a significant difference in probe-1 activity in WT and GzmA^KO^ mice, although the differences were less pronounced than in the case of tissue/cell samples (5A) suggesting the presence of other proteases in serum that could process probe-1. This result agrees with our data showing that probe-1 is also cleaved by proteases like plasmin, trypsin, and kallikrein all of which have been shown to be released in serum during inflammatory processes.^32−34^

### Sensitivity and Selectivity of GzmA Activity Detection by Combining Specific mAb and Probe-1

**Figure 7:**
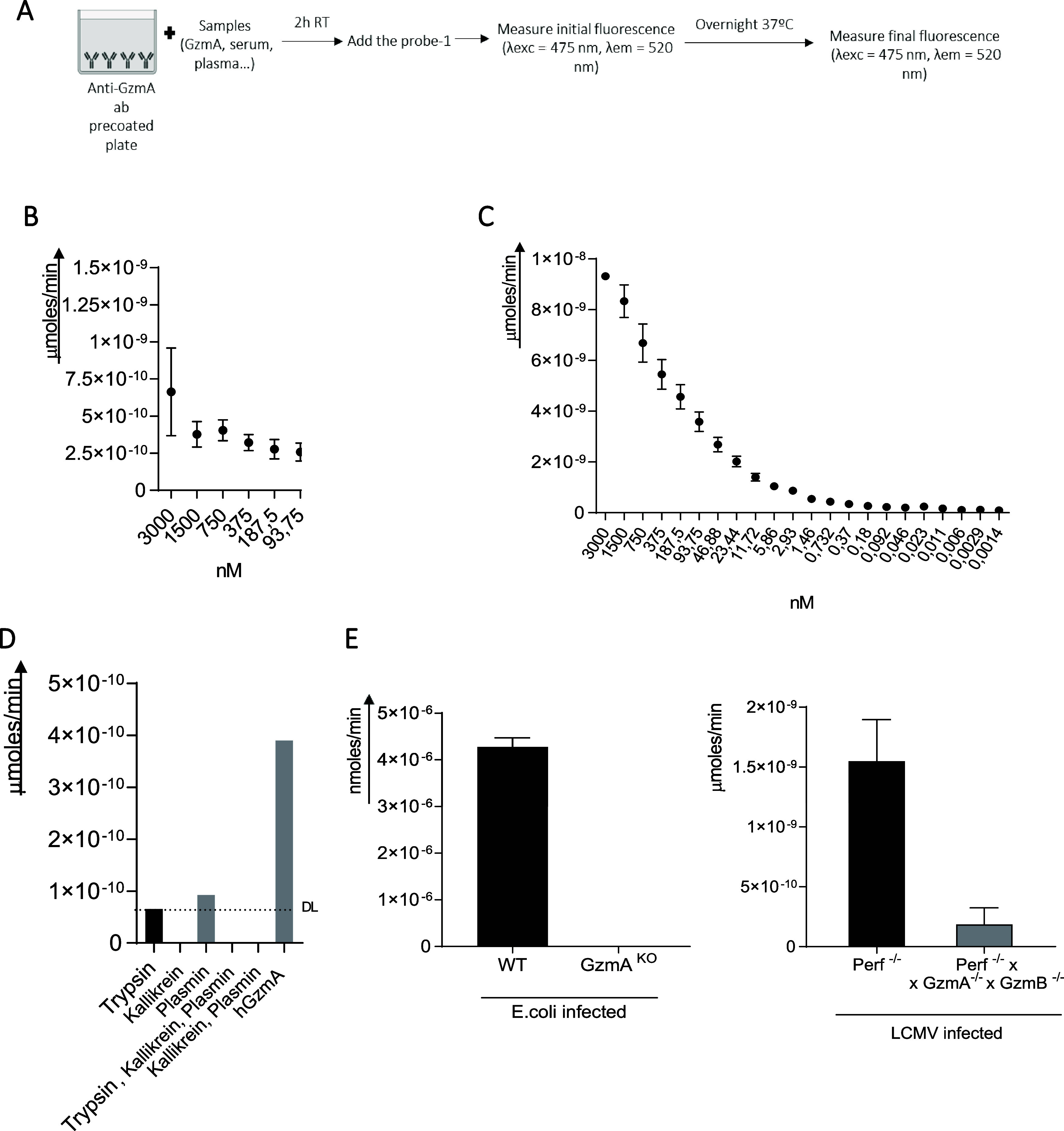
Sensitivity and selectivity of the GzmA immunoprobe (GzmA mAb + probe-1) for human and mouse GzmA. (A) Diagram of the GzmA immunoprobe procedure. (B) Different concentrations (from 3000 to 93.75 nM) of hGzmA were incubated 2 h in an anti-GzmA ab precoated plate as described in Materials and Methods. The fluorescence (λ_exc_ = 475 nm, λ_em_ = 520 nm) intensity was measured at time zero and after an overnight incubation with probe-1 (1 μM). At each of the values obtained, the signal at time 0 h and the probe-1 signal were subtracted. Enzyme activity values were calculated by extrapolating fluorescence units on a FAM calibration curve. Data are presented as mean values ± SEM of seven independent experiments. (C) Same experiment as in (A) was performed but using different concentrations (from 3000 to 0.0014 nM) of mGzmA and mGzmA Ab. Data are presented as mean values ± SEM of 10 independent experiments. (D) Same experiment as in A was performed but using trypsin (0.4 nM), kallikrein (3.7 nM), plasmin (4.3 nM) and following combinations, in where each protease is at the same concentration as individually: kallikrein and plasmin; kallikrein, plasmin and trypsin. Data are presented as mean values ± SEM of two replicates. (E) C57Bl/6 (B6) WT or GzmA^KO^ mice were inoculated with 2 × 10^8^ CFU of *E. coli* intraperitoneally and Perf^KO^ or Perf^KO^ × GzmA^KO^ × GzmB^KO^ mice were inoculated with 10^5^ pfu of LCMV intraperitoneally according to established protocols.^36^ After 24 h of sepsis induction or 8 days of HLH induction (LCMV) plasma was collected and incubated for 2 h in an anti-GzmA ab precoated plate as described in Materials and Methods. The fluorescence (λ_exc_ = 475 nm, λ_exc_ = 520 nm) intensity was measured at time zero and after overnight incubation with probe-1 (1 μM). At each of the values obtained, the signal at time 0 h and the probe-1 signal were subtracted. Enzyme activity values were calculated by extrapolating fluorescence units on a FAM calibration curve. Data are presented as mean values ± SEM of six different mice.

Although probe-1 showed good selectivity to detect GzmA activity in tissue/cell samples, in order to use it to detect GzmA activity in serum and plasma samples, we decided to develop a system combining a GzmA-specific monoclonal antibody (mAb) with probe-1 (GzmA immunoprobe; Figure 7A). After adding GzmA to mAb-coated plates, probe-1 was added, and fluorescence was measured at different time points. As shown in Figure 7, this approach showed a very good sensitivity since as low as 93.75 nM of hGzmA (Figure 7B) and 0.732 nM of mGzmA (Figure 7C) were detected.

In addition, it was shown that the signal induced by the proteases that cleaved probe-1 (trypsin, plasmin, and kallikrein) was significantly (almost completely) reduced using the GzmA immunoprobe (Figure 7D).

To further confirm the selectivity of the GzmA immunoprobe, we analyzed activity in plasma samples from WT and GzmA^KO^ mice in two different inflammatory disease models, sepsis induced by *E. coli* and familial hemophagocytic lymphohistiocytosis (fHLH). In the later model, Perforin (*Perf*) deficient mice were used to mimic human type 2 fHLH caused by mutations in the *Perf* gene.^35^

As shown in Figure 7E, the signal obtained with the GzmA immunoprobe in plasma from WT or Perf^KO^ mice was almost completely depleted in plasma from GzmA^KO^ or PerfxGzmAxB^KO^ mice. In the case of the fHLH model, we employed PerfxGzmAxB^KO^ mice, available in our lab, to compare with the samples obtained from mice deficient in *Perf*.

### Analysis of Human Fluid Samples with a GzmA Immunoprobe

**Figure 8:**
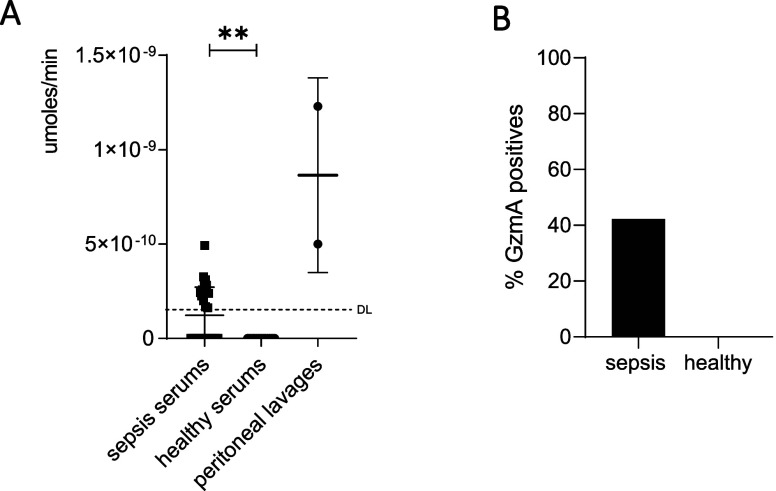
GzmA activity in sera and peritoneal lavages of healthy donors (HDs) and septic patients. (A) Serum and peritoneal lavage of septic patients and serums for healthy patients were incubated for 2 h in an anti-GzmA ab precoated plate as described in Materials and Methods. The fluorescence (λ_exc_ = 475 nm, λ_em_ = 520 nm) intensity was measured at time zero and after an overnight incubation with probe-1 (1 μM). At each of the values obtained, the signal at time 0 h, the probe-1 signal without samples, and the samples signal without probe-1 were subtracted. Enzyme activity values were calculated by extrapolating fluorescence units on a FAM calibration curve. Data are presented as mean values ± SEM of 12 sera and 2 peritoneal lavages run in duplicates. DL: detection limit. (B) Percentage of GzmA positives in septic and HDs sera analyzed in Figure 8A.

Once we had confirmed the specificity of the GzmA immunoprobe in human and mouse models, we analyzed if the GzmA immunoprobe could be used to detect extracellular GzmA activity in human samples. To this aim, we used serum and peritoneal lavage samples from patients diagnosed with sepsis and healthy donors (HD; only serum). As shown in Figure 8, GzmA activity was detected in both serum and peritoneal lavage from septic patients, while it was absent in all HDs. This result confirms that the GzmA immunoprobe can be used for the detection of extracellular GzmA in biological fluids from both mice and humans suffering from inflammatory diseases.

## Discussion

Gzms have been found in extracellular fluids of humans with different pathologies and recent evidence obtained using experimental models has shown that these extracellular Gzms are involved in the pathophysiology of different inflammatory diseases.^4^ For example, GzmB is involved in coagulation, skin aging, integrity of cellular barrier and so on.^6,7^ Extracellular GzmA has been shown to regulate inflammation in different cell types^9−11^ and it is involved in diverse inflammatory conditions including sepsis, colitis or rheumatoid arthritis.^12,14,15^ Herein, we have developed a FRET-based immunoprobe that is able to detect extracellular mouse and human GzmA activity with high selectivity and sensitivity in both inflammatory mouse experimental models and human disease. This system complements other probes previously shown to detect intracellular GzmA activity in effector and target cell^29−31^ and, fills a gap in the field allowing monitor specifically extracellular GzmA activity in inflammatory diseases.

Our results have important implications both for increasing our understanding of the biology of extracellular GzmA and for its potential application to human disease diagnosis and/or prognosis. Previous results found that in vitro GzmA did not require enzymatic activity to promote inflammation in macrophages/monocytes.^37^ In contrast, several independent groups have shown that only active GzmA is able to promote inflammation in different cell types including human and mouse macrophages/monocytes,^10,15,25,38^ epithelial and fibroblast cells.^11^ In addition, inhibition of enzyme activity in vivo reduces inflammation and improves different inflammatory diseases like sepsis,^12,25^ colitis, and colitis-induced colorectal cancer.^15^ Supporting all these results it was recently found that only active GzmA was able to induce inflammation and foot swelling when inoculated subcutaneously in mice.^39^ The immunoprobe developed here confirms that extracellular active GzmA is present in the circulation and other fluids during different inflammatory conditions in mice and humans, supporting the role of active GzmA in these diseases and endorsing the relevance of this new immunoprobe to detect active GzmA in these and likely other diseases.

The use of optical probes, including FRET-based ones, in diagnostics of different pathologies has increased in recent years due to the special characteristics of these probes including high sensitivity, specificity, short time analysis, capacity for inclusion in integrated systems, ease of automation, versatility and low price.^20^ In our case a FRET probe to detect GzmA activity was designed with a peptide sequence previously identified to be cleft by GzmA.^26^ Despite the ability of this probe to detect GzmA and its high specificity in cell/tissue lysates as found when comparing WT and KO mice, our results show that the ability of this type of probe to detect specifically GzmA activity depends on the context of application; especially, on the protease profile expressed in the cell/tissue/fluids to be analyzed. This is exemplified by our findings showing that although other members of the Gzm family like GzmB or K do not cleave probe-1, other proteases with similar catalytic activity such as plasmin, trypsin, and kallikrein, which are present in serum and other extracellular fluids, efficiently cleave probe-1 and, thus, might interfere with selective GzmA detection. Thus, we have further used probe-1 to develop a FRET-based immunoprobe with high sensitivity and specificity to detect mouse and human extracellular GzmA. This system consists of a capture antibody against GzmA followed by incubation with probe-1, which presents a high specificity to determine the enzymatic activity of GzmA, as validated using pure proteases and samples from GzmA^KO^ mice suffering from different inflammatory diseases like sepsis and HLH. The immunoprobe is also able to detect extracellular GzmA activity in human samples from septic patients. Indeed, the presence of active gzmA in serum from septic patients is significantly higher than that in HDs, confirming the potential role of active gzmA in sepsis. The new technology is affordable and accessible to any laboratory, as it relies on the use of conventional equipment, and peptides can be easily synthesized and tailored to specific needs because they are based on public sequences.

## Conclusions

To the best of our knowledge, a similar fluorescence-based assay has not been previously described and validated, and thus our results provide a new way to detect extracellular GzmA activity in mouse and human diseases. In light of the increasing evidence on the role of extracellular GzmA in inflammatory diseases, this immunoprobe provides a new tool to study the biological relevance of extracellular GzmA in the pathophysiology of inflammatory diseases, as well as screening potential GzmA inhibitors. Finally, and pending future clinical studies to validate it in proper patient cohorts, we anticipate a great potential to use this and other similar immunoprobes to help in the diagnosis and prognosis of inflammatory diseases of high relevance and socioeconomic impact.

## Supporting Information Available

Concentration of trypsin-like proteases presenting similar enzyme activity using a common substrate (PDF)

## References

[ref1] Arias M.; Martinez-Lostao L.; Santiago L.; Ferrandez A.; Granville D. J.; Pardo J. The Untold Story of Granzymes in Oncoimmunology: Novel Opportunities with Old Acquaintances. Trends cancer 2017, 3, 407–422. 10.1016/j.trecan.2017.04.001. 28718416

[ref2] Pardo J.; Aguilo J. I.; Anel A.; Martin P.; Joeckel L.; Borner C.; Wallich R.; Mullbacher A.; Froelich C. J.; Simon M. M. The biology of cytotoxic cell granule exocytosis pathway: granzymes have evolved to induce cell death and inflammation. Microbes Infect. 2009, 11, 452–459. 10.1016/j.micinf.2009.02.004. 19249384

[ref3] Voskoboinik I.; Whisstock J. C.; Trapani J. A. Perforin and granzymes: function, dysfunction and human pathology. Nat. Rev. Immunol. 2015, 15, 388–400. 10.1038/nri3839. 25998963

[ref4] Richardson K. C.; Jung K.; Pardo J.; Turner C. T.; Granville D. J. Noncytotoxic Roles of Granzymes in Health and Disease. Physiology (Bethesda) 2022, 37, 323–348. 10.1152/physiol.00011.2022. 35820180

[ref5] Ramírez-Labrada A.; Pesini C.; Santiago L.; Hidalgo S.; Calvo-Pérez A.; Oñate C.; Andrés-Tovar A.; Garzón-Tituaña M.; Uranga-Murillo I.; Arias M. A.; Galvez E. M.; Pardo J. All About (NK Cell-Mediated) Death in Two Acts and an Unexpected Encore: Initiation, Execution and Activation of Adaptive Immunity. Front. Immunol. 2022, 13, 896228 10.3389/fimmu.2022.896228. 35651603 PMC9149431

[ref6] Hiebert P. R.; Wu D.; Granville D. J. Granzyme B degrades extracellular matrix and contributes to delayed wound closure in apolipoprotein E knockout mice. Cell Death Differ. 2013, 20, 1404–1414. 10.1038/cdd.2013.96. 23912712 PMC3770318

[ref7] Pardo J.; Wallich R.; Ebnet K.; Iden S.; Zentgraf H.; Martin P.; Ekiciler A.; Prins A.; Mullbacher A.; Huber M.; et al. Granzyme B is expressed in mouse mast cells in vivo and in vitro and causes delayed cell death independent of perforin. Cell Death Differ. 2007, 14, 1768–1779. 10.1038/sj.cdd.4402183. 17599099

[ref8] Garzón-Tituaña M.; Sierra-Monzón J. L.; Comas L.; Santiago L.; Paño-Pardo J. R.; Galvez E. M.; Pardo J.; Arias M. Granzyme A inhibition reduces inflammation and increases survival during abdominal sepsis. Theranostics 2020, 11, 3781–3795. 10.7150/thno.49288. PMC7914344 33664861

[ref9] Metkar S. S.; Menaa C.; Pardo J.; Wang B.; Wallich R.; Freudenberg M.; Kim S.; Raja S. M.; Shi L.; Simon M. M.; et al. Human and mouse granzyme A induce a proinflammatory cytokine response. Immunity 2008, 29, 720–733. 10.1016/j.immuni.2008.08.014. 18951048

[ref10] Sower L. E.; Froelich C. J.; Allegretto N.; Rose P. M.; Hanna W. D.; Klimpel G. R. Extracellular activities of human granzyme A. Monocyte activation by granzyme A versus alpha-thrombin. J. Immunol. 1996, 156, 2585–2590. 10.4049/jimmunol.156.7.2585. 8786323

[ref11] Sower L. E.; Klimpel G. R.; Hanna W.; Froelich C. J. Extracellular activities of human granzymes. I. Granzyme A induces IL6 and IL8 production in fibroblast and epithelial cell lines. Cell. Immunol. 1996, 171, 159–163. 10.1006/cimm.1996.0187. 8754861

[ref12] Uranga-Murillo I.; Tapia E.; Garzon-Tituana M.; Ramirez-Labrada A.; Santiago L.; Pesini C.; Esteban P.; Roig F. J.; Galvez E. M.; Bird P. I.; et al. Biological relevance of Granzymes A and K during E. coli sepsis. Theranostics 2021, 11, 9873–9883. 10.7150/thno.59418. 34815792 PMC8581435

[ref13] Anthony D. A.; Andrews D. M.; Chow M.; Watt S. V.; House C.; Akira S.; Bird P. I.; Trapani J. A.; Smyth M. J. A role for granzyme M in TLR4-driven inflammation and endotoxicosis. J. Immunol. 2010, 185, 1794–1803. 10.4049/jimmunol.1000430. 20585036

[ref14] Santiago L.; Menaa C.; Arias M.; Martin P.; Jaime-Sánchez P.; Metkar S.; Comas L.; Erill N.; Gonzalez-Rumayor V.; Esser E.; Galvez E. M.; Raja S.; Simon M. M.; Sprague S. M.; Gabay C.; Martinez-Lostao L.; Pardo J.; Froelich C. J. Granzyme A Contributes to Inflammatory Arthritis in Mice Through Stimulation of Osteoclastogenesis. Arthritis Rheumatol. 2017, 69, 320–334. 10.1002/art.39857. 27598995

[ref15] Santiago L.; Castro M.; Sanz-Pamplona R.; Garzon M.; Ramirez-Labrada A.; Tapia E.; Moreno V.; Layunta E.; Gil-Gomez G.; Garrido M.; et al. Extracellular Granzyme A Promotes Colorectal Cancer Development by Enhancing Gut Inflammation. Cell Rep. 2020, 32, 107847 10.1016/j.celrep.2020.107847. 32640217

[ref16] Buzza M. S.; Bird P. I. Extracellular granzymes: current perspectives. Biol. Chem. 2006, 387, 827–837. 10.1515/BC.2006.106. 16913832

[ref17] de Jong H. K.; Garcia-Laorden M. I.; Hoogendijk A. J.; Parry C. M.; Maude R. R.; Dondorp A. M.; Faiz M. A.; van der Poll T.; Wiersinga W. J. Expression of intra- and extracellular granzymes in patients with typhoid fever. PLoS Neglected Trop. Dis. 2017, 11, e0005823 10.1371/journal.pntd.0005823. PMC5549753 28749963

[ref18] Mackie E. J.; Pagel C. N.; Smith R.; de Niese M. R.; Song S. J.; Pike R. N. Protease-activated receptors: a means of converting extracellular proteolysis into intracellular signals. IUBMB Life 2002, 53, 277–281. 10.1080/15216540213469. 12625364

[ref19] Pryzdial E. L. G.; Leatherdale A.; Conway E. M. Coagulation and complement: Key innate defense participants in a seamless web. Front. Immunol. 2022, 13, 918775 10.3389/fimmu.2022.918775. 36016942 PMC9398469

[ref20] Ong I. L. H.; Yang K. L. Recent developments in protease activity assays and sensors. Analyst 2017, 142, 1867–1881. 10.1039/C6AN02647H. 28487913

[ref21] Jares-Erijman E. A.; Jovin T. M. FRET imaging. Nat. Biotechnol. 2003, 21, 1387–1395. 10.1038/nbt896. 14595367

[ref22] Scott J. I.; Mendive-Tapia L.; Gordon D.; Barth N. D.; Thompson E. J.; Cheng Z.; Taggart D.; Kitamura T.; Bravo-Blas A.; Roberts E. W.; Juarez-Jimenez J.; Michel J.; Piet B.; de Vries I. J.; Verdoes M.; Dawson J.; Carragher N. O.; Connor R. A. O.; Akram A. R.; Frame M.; Vendrell M. A fluorogenic probe for granzyme B enables in-biopsy evaluation and screening of response to anticancer immunotherapies. Nat. Commun. 2022, 13, 2366 10.1038/s41467-022-29691-w. 35501326 PMC9061857

[ref23] Pardo J.; Wallich R.; Martin P.; Urban C.; Rongvaux A.; Flavell R. A.; Müllbacher A.; Borner C.; Simon M. M. Granzyme B-induced cell death exerted by ex vivo CTL: discriminating requirements for cell death and some of its signs. Cell Death Differ. 2008, 15, 567–579. 10.1038/sj.cdd.4402289. 18064039

[ref24] Catalán E.; Jaime-Sánchez P.; Aguiló N.; Simon M. M.; Froelich C. J.; Pardo J. Mouse cytotoxic T cell-derived granzyme B activates the mitochondrial cell death pathway in a Bim-dependent fashion. J. Biol. Chem. 2015, 290, 6868–6877. 10.1074/jbc.M114.631564. 25605735 PMC4358112

[ref25] Garzon-Tituana M.; Sierra-Monzon J. L.; Comas L.; Santiago L.; Khaliulina-Ushakova T.; Uranga-Murillo I.; Ramirez-Labrada A.; Tapia E.; Morte-Romea E.; Algarate S.; et al. Granzyme A inhibition reduces inflammation and increases survival during abdominal sepsis. Theranostics 2021, 11, 3781–3795. 10.7150/thno.49288. 33664861 PMC7914344

[ref26] Kaiserman D.; Stewart S. E.; Plasman K.; Gevaert K.; Van Damme P.; Bird P. I. Identification of Serpinb6b as a species-specific mouse granzyme A inhibitor suggests functional divergence between human and mouse granzyme A.*J*. Biol. Chem. 2014, 289, 9408–9417. 10.1074/jbc.M113.525808. PMC3979379 24505135

[ref27] Fan Z.; Beresford P. J.; Zhang D.; Lieberman J. HMG2 interacts with the nucleosome assembly protein SET and is a target of the cytotoxic T-lymphocyte protease granzyme A. Mol. Cell. Biol. 2002, 22, 2810–2820. 10.1128/MCB.22.8.2810-2820.2002. 11909973 PMC133744

[ref28] Bell J. K.; Goetz D. H.; Mahrus S.; Harris J. L.; Fletterick R. J.; Craik C. S. The oligomeric structure of human granzyme A is a determinant of its extended substrate specificity. Nat. Struct. Biol. 2003, 10, 527–534. 10.1038/nsb944. 12819769

[ref29] Vrazo A. C.; Hontz A. E.; Figueira S. K.; Butler B. L.; Ferrell J. M.; Binkowski B. F.; Li J.; Risma K. A. Live cell evaluation of granzyme delivery and death receptor signaling in tumor cells targeted by human natural killer cells. Blood 2015, 126, e1–e10. 10.1182/blood-2015-03-632273. 26124495 PMC4543232

[ref30] Kołt S.; Janiszewski T.; Kaiserman D.; Modrzycka S.; Snipas S. J.; Salvesen G.; Dra G. M.; Bird P. I.; Kasperkiewicz P. Detection of Active Granzyme A in NK92 Cells with Fluorescent Activity-Based Probe. J. Med. Chem. 2020, 63, 3359–3369. 10.1021/acs.jmedchem.9b02042. 32142286 PMC7590976

[ref31] Cheng Z.; Thompson E. J.; Mendive-Tapia L.; Scott J. I.; Benson S.; Kitamura T.; Senan-Salinas A.; Samarakoon Y.; Roberts E. W.; Arias M. A.; Pardo J.; Galvez E. M.; Vendrell M. Fluorogenic Granzyme A Substrates Enable Real-Time Imaging of Adaptive Immune Cell Activity. Angew. Chem. 2023, 62, e202216142 10.1002/anie.202216142. 36562327 PMC10108010

[ref32] Huang W. C.; Chuang C. F.; Huang Y. T.; Chung I. C.; Chen M. L.; Chuang T. Y.; Yang X. L.; Chou Y. Y.; Liu C. H.; Chen N. Y.; Chen C. J.; Yuan T. T. Monoclonal enolase-1 blocking antibody ameliorates pulmonary inflammation and fibrosis. Respir. Res. 2023, 24, 280 10.1186/s12931-023-02583-3. 37964270 PMC10647181

[ref33] Liu K.; Liu J.; Zou B.; Li C.; Zeh H. J.; Kang R.; Kroemer G.; Huang J.; Tang D. Trypsin-Mediated Sensitization to Ferroptosis Increases the Severity of Pancreatitis in Mice. Cell. Mol. Gastroenterol.Hepatol. 2022, 13, 483–500. 10.1016/j.jcmgh.2021.09.008. 34562639 PMC8688567

[ref34] Motta G.; Juliano L.; Chagas J. R. Human plasma kallikrein: roles in coagulation, fibrinolysis, inflammation pathways, and beyond. Front. Physiol. 2023, 14, 1188816 10.3389/fphys.2023.1188816. 37711466 PMC10499198

[ref35] Voskoboinik I.; Trapani J. A. Perforinopathy: a spectrum of human immune disease caused by defective perforin delivery or function. Front. Immunol. 2013, 4, 441 10.3389/fimmu.2013.00441. 24376445 PMC3860100

[ref36] Pardo J.; Balkow S.; Anel A.; Simon M. M. The differential contribution of granzyme A and granzyme B in cytotoxic T lymphocyte-mediated apoptosis is determined by the quality of target cells. Eur. J. Immunol. 2002, 32, 1980–1985. 10.1002/1521-4141(200207)32:7<1980::AID-IMMU1980>3.0.CO;2-Z. 12115618

[ref37] Wensink A. C.; Kok H. M.; Meeldijk J.; Fermie J.; Froelich C. J.; Hack C. E.; Bovenschen N. Granzymes A and K differentially potentiate LPS-induced cytokine response. Cell death discovery 2016, 2, 16084 10.1038/cddiscovery.2016.84. 28028441 PMC5149580

[ref38] Campbell R. A.; Franks Z.; Bhatnagar A.; Rowley J. W.; Manne B. K.; Supiano M. A.; Schwertz H.; Weyrich A. S.; Rondina M. T. Granzyme A in Human Platelets Regulates the Synthesis of Proinflammatory Cytokines by Monocytes in Aging. J. Immunol. 2018, 200, 295–304. 10.4049/jimmunol.1700885. 29167233 PMC5736423

[ref39] Schanoski A. S.; Le T. T.; Kaiserman D.; Rowe C.; Prow N. A.; Barboza D. D.; Santos C. A.; Zanotto P. M. A.; Magalhaes K. G.; Aurelio L.; et al. Granzyme A in Chikungunya and Other Arboviral Infections. Front. Immunol. 2020, 10, 3083 10.3389/fimmu.2019.03083. 31993061 PMC6971054

