# Supplementary material for: Selective Detection of Active Extracellular Granzyme A by Using a Novel Fluorescent Immunoprobe with Application to Inflammatory Diseases
Source: ACS Pharmacol Transl Sci. 2024 Apr 22;7(5):1474–84. doi: 10.1021/acsptsci.4c00065 (PMC11092195; doi:10.1021/acsptsci.4c00065)
Supplement: Supplementary file 1 — pt4c00065_si_001.pdf [file pt4c00065_si_001.pdf]

SUPPORTING INFORMATION

Selective detection of active extracellular granzyme A by using a novel fluorescent immunoprobe with application to inflammatory diseases

Ana Senan-Salinas[1#], Laura Comas[1#], Patricia Esteban[2], Marcela Garzón-Tituaña[3,4], Zhiming Cheng[6,8], Llipsy Santiago[1], Maria Pilar Domingo[1], Ariel Ramírez-Labrada[2,4,7], José Ramón Paño-Pardo[4,5], Marc Vendrell[6], Julián Pardo[2,3,4], Maykel A Arias[2,4*] and Eva M Galvez[1,4 *]

[1]Instituto de Carboquímica ICB-CSIC, 50018, Zaragoza, Spain.

[2]Fundación Instituto de Investigación Sanitaria Aragón (IIS Aragón), BiomedicalResearch Centre of Aragón (CIBA), 50009, Zaragoza, Spain.

[3]Dept. Microbiology, Preventive Medicine and Public Health, University of Zaragoza, 50009, Zaragoza, Spain.

[4] CIBERINFEC, ISCIII - CIBER de Enfermedades Infecciosas, Instituto de Salud Carlos III, Madrid, Spain

[5]Servicio de Enfermedades Infecciosas, Hospital Clinico Universitario Lozano Blesa, Zaragoza, 50009, Spain

[6]Centre for Inflammation Research,The University of Edinburgh, EH164UU Edinburgh, U.K

[7]Unidad de Nanotoxicología e Inmunotoxicología (UNATI), Centro de Investigación Biomédica de Aragón (CIBA), Aragón HealthResearchInstitute (IIS Aragón), Zaragoza, Spain

[8]IRR Chemistry Hub, Institute for Regeneration and Repair, The University of Edinburgh, EH16 4UU Edinburgh, U.K.

[#] Authors contribute equally

*Corresponding Authors:

Eva M Galvez, Instituto de Carboquímica ICB-CSIC, 50018. Zaragoza, Spain; e-mail: eva@icb.csic.es ;

Maykel A Arias, Fundación Instituto de Investigación Sanitaria Aragón (IIS Aragón), BiomedicalResearch Centre of Aragón (CIBA), 50009, Zaragoza, Spain.e-mail: maykelariascabrero@gmail.com

Table of contents for the supporting information

1. **Supplementary table 1. Concentration of trypsin-like proteases presenting similar enzyme activity using a common substrate.**

Table S1

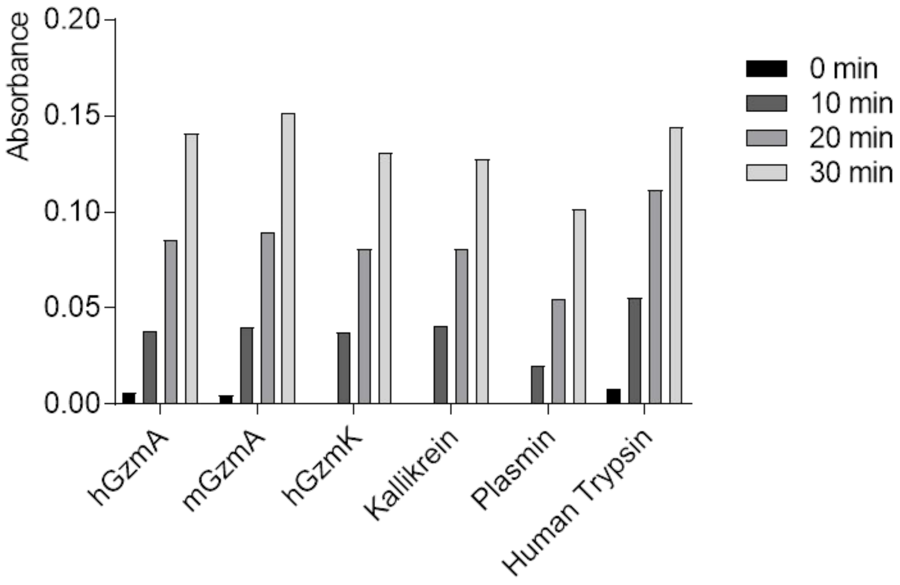

Table S1. Concentration of trypsin-like proteases presenting similar enzyme activity using a common substrate. The substrate Nα-CBZ-L-Lysine thiobenzyl ester hydrochloride was incubated with hGzmA (66.7 nM), hGzmK (2.4 nM), mGzmA (4 nm), Kallikrein (3.7 nM), Plasmin (4.3 nM) and Trypsin (0.4 nM) for 30 min as described in methods. The absorbance was measured. The values obtained at each time were subtracted from the substrate signal in the absence of enzymes. Data represent the mean of three independent replicates.
